# Association of Baseline and Dynamic Changes in Growth Differentiation Factor-15 Changes with Longitudinal Myocardial Function in Sepsis

**DOI:** 10.3390/jcdd13080391

**Published:** 2026-08-14

**Authors:** Ivana Lukić, Damir Mihić, Kristina Selthofer-Relatić, Ivana Arambašić, Jerko Barbić, Sanja Mandić, Silvija Canecki-Varžić, Lana Maričić

**Affiliations:** 1 Department of Cardiology, Clinical Hospital Center Osijek, 31000 Osijek, Croatia; 2 Faculty of Medicine Osijek, J.J. Strossmayer University Osijek, 31000 Osijek, Croatia; 3 Department of Intensive Care for Internal Medicine, Clinic of Internal Medicine, Clinical Hospital Center Osijek, 31000 Osijek, Croatia; 4 Department of Nephrology, University Hospital Centre Osijek, 31000 Osijek, Croatia; 5 Department of Endocrinology, Clinical Hospital Center Osijek, 31000 Osijek, Croatia

**Keywords:** sepsis, echocardiography, cardiomyopathies, troponin I, growth differentiation factor 15

## Abstract

Septic cardiomyopathy is a common complication of sepsis that significantly contributes to hemodynamic instability and adverse clinical outcomes. Despite increasing understanding of its pathophysiology, there is still no specific biomarker that reliably reflects myocardial function during sepsis. Growth differentiation factor-15 (GDF-15) is a cytokine induced by cellular stress associated with inflammation, oxidative stress, and cardiovascular damage. However, the relationship between GDF-15 and echocardiographic markers of septic myocardial dysfunction remains poorly understood. The aim of this study was to examine the association of serum concentrations of GDF-15 and high-sensitivity troponin I (hs-TnI) with echocardiographic parameters of systolic and diastolic myocardial function in patients with sepsis. This prospective observational study included 50 patients with sepsis treated in the Intensive Care Unit of the Osijek Clinical Hospital Center between 2023 and 2025. GDF-15 and hs-TnI concentrations were measured at admission and during follow-up, while transthoracic echocardiography was performed at the same time points to assess myocardial function. Single concentrations of GDF-15 were not significantly associated with most conventional echocardiographic parameters, but showed a positive association with the E/A ratio. In multivariable analysis adjusted for illness severity (SOFA score), admission GDF-15 was independently associated with left ventricular longitudinal systolic function (MAPSE). Changes in GDF-15 during hospitalization were associated with echocardiographic measurements of longitudinal myocardial function obtained at admission and follow-up, including mitral annular plane systolic excursion (MAPSE), tricuspid annular plane systolic excursion (TAPSE), tissue Doppler systolic velocity (S′), and isovolumetric relaxation time (IVRT). In contrast, hs-TnI did not show a consistent association with echocardiographic parameters. These findings suggest that serial GDF-15 assessment may provide additional insight into myocardial functional changes during sepsis and complement the echocardiographic evaluation of septic cardiomyopathy.

## 1. Introduction

Sepsis is a life-threatening organ dysfunction caused by a dysregulated host response to infection and is a leading cause of mortality and burden on healthcare systems in intensive care units worldwide [1,2]. Despite significant progress in understanding the pathophysiology of sepsis, early recognition of the disease, and modern therapeutic strategies, mortality in patients with severe sepsis and septic shock remains high, especially in the presence of multiorgan dysfunction [3].

Cardiovascular dysfunction is one of the most important manifestations of sepsis and significantly contributes to hemodynamic instability, disruption of tissue perfusion and unfavorable clinical outcomes [4]. There is increasing evidence that myocardial dysfunction is not only a consequence of advanced sepsis, but an active pathobiological process that participates in the progression of organ failure and contributes to increased mortality. Consequently, septic cardiomyopathy has become one of the most intensively researched areas of modern intensive care and cardiovascular medicine.

Septic cardiomyopathy is now considered a complex, heterogeneous, and potentially reversible syndrome characterized by acute dysfunction of systolic and/or diastolic myocardial function in the absence of primary coronary artery disease as the cause of heart failure [5,6]. The pathophysiological basis includes activation of inflammatory cytokines, oxidative and nitrosamine stress, endothelial dysfunction, microcirculatory dysfunction, mitochondrial dysfunction, alterations in calcium signaling, and metabolic reprogramming of cardiomyocytes [7,8]. These mechanisms lead to disruption of myocardial contractility, relaxation, and energy homeostasis, creating a characteristic but highly dynamic phenotype of cardiac dysfunction during sepsis [9].

In recent years, there has been increasing evidence that septic cardiomyopathy does not represent a single clinical entity, but rather a spectrum of different phenotypes of myocardial dysfunction that differ according to the dominant involvement of the left or right ventricle, the presence of systolic or diastolic dysfunction, and the time course of recovery [10]. Recent reviews emphasize that the heterogeneity of clinical presentation and the lack of standardized diagnostic criteria represent a major limitation to a better understanding of the pathophysiology and the development of targeted therapeutic approaches to septic cardiomyopathy. This understanding further emphasizes the need to identify new biomarkers that could more accurately reflect the presence and severity of myocardial dysfunction during sepsis.

One of the greatest challenges in the current approach to septic cardiomyopathy is the fact that there is no single diagnostic criterion or specific biomarker that would reliably identify the presence and severity of myocardial dysfunction. Echocardiography therefore represents the fundamental method for assessing cardiac function in patients with sepsis because it allows noninvasive assessment of structural and functional changes in the myocardium in real time. However, conventional parameters, especially left ventricular ejection fraction (LVEF), often do not reflect the true degree of myocardial damage due to their pronounced dependence on preload, afterload and vasopressor support [11].

For this reason, increasing attention is directed towards more sensitive indicators of longitudinal myocardial function, including mitral annular plane systolic excursion (MAPSE), tricuspid annular plane systolic excursion (TAPSE), tissue Doppler systolic wave (S′) and global longitudinal strain (GLS), which can detect subclinical myocardial dysfunction even in patients with preserved ejection fraction [12]. Studies based on speckle-tracking echocardiography have shown that impairment of longitudinal myocardial function is associated with disease severity and adverse clinical outcomes even when the ejection fraction is preserved [13]. In addition to systolic dysfunction, there is increasing evidence that diastolic dysfunction is an important prognostic factor in patients with severe sepsis and septic shock [14].

Biomarkers play an increasingly important role in assessing disease severity, organ dysfunction and prognosis in patients with sepsis. Growth differentiation factor-15 (GDF-15) is a member of the transforming growth factor beta (TGF-β) superfamily and is a stress-induced cytokine whose expression significantly increases under conditions of inflammation, hypoxia, oxidative stress and tissue damage [15]. In the last decade, GDF-15 has attracted considerable attention as an integrative biomarker that reflects the total biological burden of the organism, including activation of inflammatory pathways, cellular stress, metabolic dysregulation, and cardiovascular damage [16].

In cardiovascular medicine, elevated GDF-15 concentrations are consistently associated with adverse outcomes in patients with heart failure, acute coronary syndrome and other forms of cardiovascular disease [17]. Unlike traditional biomarkers that reflect specific pathophysiological processes, such as cardiomyocyte necrosis or hemodynamic load, GDF-15 integrates information about multiple interrelated damage mechanisms, including inflammation, oxidative stress, mitochondrial dysfunction, and cellular stress response [18]. These mechanisms are among the key drivers of the development of septic cardiomyopathy, which makes GDF-15 a particularly interesting candidate for the assessment of myocardial dysfunction in sepsis.

In patients with sepsis, GDF-15 has been primarily investigated as a prognostic biomarker. GDF-15 concentrations have been shown to be significantly elevated in critically ill patients and to correlate with the severity of the disease, the degree of organ dysfunction, and an increased risk of death [19]. However, despite the growing body of research, most of the available data focus on the prognostic value of GDF-15, while its relationship to objective indicators of cardiac function remains poorly understood.

The relationship between GDF-15 and septic cardiomyopathy is of particular interest. Activation of inflammatory signaling pathways, mitochondrial dysfunction, oxidative stress and disruption of energy homeostasis represent common pathophysiological mechanisms that simultaneously promote the development of myocardial dysfunction and GDF-15 expression [10]. Experimental studies have shown that GDF-15 participates in the regulation of myocardial damage and the adaptive response to acute stress and can have a cardioprotective role by modulating inflammation, oxidative stress and cell death [20]. Furthermore, recent experimental models of sepsis have shown that GDF-15 can alleviate sepsis-induced myocardial dysfunction by inhibiting cardiomyocyte ferroptosis via the SOCS1/GPX4 signaling pathway, further confirming its potential involvement in the mechanisms of septic cardiomyopathy [21].

Despite these findings, clinical data on the association of GDF-15 with echocardiographic markers of myocardial function in sepsis are extremely limited. Previous studies suggest that GDF-15 represents an integrative biomarker of systemic and cardiovascular stress, but it remains unclear whether its concentration reflects only the severity of systemic disease or also specific processes associated with the development of septic cardiomyopathy. Since septic cardiomyopathy is considered a dynamic syndrome whose functional characteristics change during the course of the disease, a single biomarker determination is probably not sufficient for an accurate assessment of myocardial dysfunction. Therefore, analysis of dynamic changes in GDF-15 could provide additional insight into the relationship between systemic cellular stress and the functional response of the myocardium during sepsis. Such an approach has been poorly investigated to date, especially in relation to longitudinal echocardiographic markers of myocardial function.

In this context, it is particularly important to compare GDF-15 with hs-TnI. Troponin I reflects cardiomyocyte damage and is often elevated in patients with sepsis, but its increase does not necessarily correspond to the degree of functional myocardial dysfunction [22]. Therefore, GDF-15, as a biomarker of systemic and myocardial stress, could provide additional information on the functional status of the myocardium that is not available using conventional biomarkers.

Accordingly, the aim of this study was to examine the association of serum GDF-15 and hs-TnI concentrations with echocardiographic parameters of systolic and diastolic myocardial function in patients with sepsis, with a special emphasis on longitudinal indicators of myocardial function and dynamic changes in biomarkers during hospitalization.

## 2. Materials and Methods

### 2.1. Participants and Study Design

A prospective cohort study was conducted at the Department of Intensive Care for Internal Medicine Patients, Clinic of Internal Medicine, Clinical Hospital Center Osijek, Croatia, between 2023 and 2025. The study included 50 hospitalized patients aged 18 years or older with sepsis or septic shock.

Sepsis was defined as life-threatening organ dysfunction caused by a dysregulated host response to infection, with organ dysfunction determined by an increase in the Sequential Organ Failure Assessment (SOFA) score of ≥2 points. Septic shock was defined as persistent hypotension that requires the use of vasopressors to maintain mean arterial pressure (MAP) ≥ 65 mmHg with a serum lactate concentration > 2 mmol/L despite adequate volume replacement [1].

Exclusion criteria were pregnancy, acute myocardial infarction type 1, acute heart failure, pulmonary embolism, active malignant disease, active oncological treatment, autoimmune diseases, and acute liver failure.

The study was conducted in accordance with the principles of the Declaration of Helsinki and was approved by the Ethics Committee of the Clinical Hospital Center Osijek (approval number: R1-1510/2023). All patients or their legal representatives provided written informed consent to participate in the study.

### 2.2. Clinical and Laboratory Data

Demographic and clinical data were collected at admission, including age, gender, source of infection, and SOFA score. Laboratory workup included routine hematological and biochemical parameters and determination of biomarkers, including high-sensitivity troponin I (hs-TnI) and growth differentiation factor-15 (GDF-15).

Peripheral venous blood samples were collected at two predefined study time points: at hospital admission and during follow-up (days 5–7 of hospitalization). After centrifugation, the serum was stored at the appropriate temperature until analysis. Serum GDF-15 concentration (pg/mL) was determined by electrochemiluminescent immunoassay (ECLIA), using the cobas e 411 analyzer (Roche Diagnostics GmbH, Mannheim, Germany, Software Version 03-01) according to the manufacturer’s instructions in a certified laboratory of the Clinical Hospital Center Osijek. Hs-TnI concentration (ng/L) was determined using an electrochemiluminescent method (Siemens Healthineers, Tarrytown, NY, USA) according to standard laboratory protocols.

### 2.3. Echocardiographic Analysis

All patients underwent transthoracic echocardiography using a Philips CX50 device at the same two predefined study time points: at hospital admission and during follow-up (days 5–7 of hospitalization). Parameters of left and right ventricular systolic and diastolic function were assessed, including left ventricular ejection fraction determined by the Simpson biplane method, MAPSE, TAPSE, transmitral flow (E and A wave), E/A ratio, tissue Doppler (E′), E/E′ ratio, IVRT, and longitudinal right ventricular systolic function (S′).

### 2.4. Statistical Analysis

Categorical variables are presented as absolute and relative frequencies, while continuous variables are presented as median and interquartile range. Normality of distribution was assessed using the Shapiro–Wilk test. Differences between groups were analyzed using the Mann–Whitney U test, while changes between two measurements were analyzed using the Wilcoxon signed-rank test, with Hodges–Lehmann estimates and 95% confidence intervals reported. The association between variables was assessed using Spearman’s rank correlation coefficient. Additionally, bivariate linear regression analysis was performed to assess the association of echocardiographic parameters with biomarker changes [23,24]. Multivariate linear regression analysis was subsequently performed to evaluate the associations of GDF-15, ΔGDF-15, hs-TnI, and SOFA score with MAPSE and TAPSE. All *p* values were two-sided. All statistical tests were two-sided, and *p* values < 0.05 were considered statistically significant. Statistical analyses were performed using MedCalc^®^ Statistical Software version 23.6.0 (MedCalc Software Ltd., Ostend, Belgium). The reporting of statistical analyses followed the SAMPL (Statistical Analyses and Methods in the Published Literature) Guidelines [25].

## 3. Results

### 3.1. General and Clinical Characteristics of the Patients

A total of 50 patients with sepsis were included in the study, of whom 28 (56%) were men and 22 (44%) were women. The median age of the patients was 71 years (interquartile range 62–80 years). The median SOFA score on admission was 8, with a total range of values from 3 to 12. The most common diagnosis was sepsis caused by pneumonia, present in 30 (60%) patients. Urosepsis was the second most common source of sepsis and was observed in 12 (24%) patients. Other causes of sepsis were significantly less common (Table 1).

### 3.2. Differences in hs-TnI and GDF-15 Values at Admission and Follow-Up Measurement

The median hs-TnI value was higher at admission compared to the follow-up measurement, as were the GDF-15 values. Both biomarkers showed considerable interindividual variability, but without statistically significant differences between measurements (Table 2).

### 3.3. Correlation of GDF-15 Serum Concentration with Echocardiographic Parameters

Echocardiographic parameters did not differ significantly between groups defined by median GDF-15 values at admission or during follow-up measurements (Table 3). Serum GDF-15 concentration at admission was positively and significantly associated with E/A ratio (Rho = 0.330), while the association with other echocardiographic parameters was not statistically significant. GDF-15 values during follow-up measurements were also positively associated with E/A ratio (Rho = 0.411). Change in GDF-15 concentration during hospitalization was negatively associated with longitudinal systolic function parameters, including MAPSE, TAPSE and S′, while a positive association was observed with IVRT during follow-up measurements (Table 4).

### 3.4. Relationship Between hs-TnI and Echocardiographic Parameters

High-sensitivity troponin I values at admission and during follow-up measurements did not show a significant correlation with most echocardiographic parameters. The change in hs-TnI during hospitalization was positively associated with E′ at admission, while the relative change in hs-TnI showed a positive correlation with E′ at admission and a negative correlation with S′ during follow-up measurements (Table 5).

### 3.5. Linear Regression Analysis

Bivariate linear regression analysis did not show a statistically significant association between echocardiographic parameters and the log-transformed change in GDF-15 (log(ΔGDF-15)), either at admission or at follow-up (Table 6). No significant associations were found between echocardiographic parameters and log-transformed hs-TnI changes (Table 7).

### 3.6. Multivariate Analysis of Association of GDF-15, hs-TnI and SOFA Score with MAPSE and TAPSE

Multivariate linear regression analysis showed a significant model for MAPSE at admission, with log-transformed GDF-15 concentration and SOFA score together explaining 12.5% of MAPSE variability. In the model, the log-transformed concentration of GDF-15 was significantly positively associated with MAPSE, while the SOFA score was not a significant predictor. For TAPSE at admission and MAPSE and TAPSE at follow-up, no statistically significant associations were found. (Table 8).

In multivariate linear regression analysis, log-transformed hs-TnI concentration and SOFA score did not show a significant association with MAPSE and TAPSE at admission or at control, and none of the regression models were significant (Table 9).

## 4. Discussion

In this prospective observational study, the association of serum concentrations of GDF-15 and hs-TnI with echocardiographic parameters of myocardial systolic and diastolic function in patients with sepsis was examined. The main finding of the study was that GDF-15 did not show a significant association with most conventional echocardiographic parameters at individual time points, while its dynamic changes during hospitalization were associated with parameters of longitudinal systolic function and myocardial diastolic relaxation. Conversely, hs-TnI did not show a consistent association with echocardiographic parameters. These findings suggest that GDF-15 in patients may reflect not only global systolic function, but also a broader biological response of the myocardium to inflammation, hypoxia, oxidative stress and hemodynamic load and support the growing concept that serial biomarker assessment may provide greater clinical insight than isolated measurements when evaluating septic cardiomyopathy [26,27]. Given the relatively small sample size, the number of covariates included in the multivariate models was intentionally restricted to reduce the risk of overfitting.

Septic cardiomyopathy is now considered a heterogeneous and dynamic syndrome, rather than a unique disorder that can be reliably defined only by left ventricular ejection fraction. Previous research and review papers emphasize that the interpretation of the ejection fraction in sepsis is limited due to the pronounced dependence on preload, afterload, vasopressor support and the overall hemodynamic status of the patient [9,11]. Recent literature further emphasizes that the absence of universally accepted diagnostic criteria remains one of the major challenges in septic cardiomyopathy, supporting the need for an integrated diagnostic approach that combines echocardiographic, clinical, and biomarker-based assessment [28]. These findings further support the concept that the ejection fraction in this group of patients must be interpreted in the context of the overall hemodynamic status of the patient. Accordingly, in our study, no significant association of GDF-15 with the ejection fraction of the left ventricle was established. Based on these findings, GDF-15 may reflect the systemic inflammatory response and myocardial injury in sepsis and the ejection fraction is not a good indicator of myocardial damage. This finding does not exclude myocardial dysfunction, but additionally supports the view that conventional parameters may be insufficiently sensitive for the detection of early and subtle functional changes of the myocardium during sepsis.

Unlike ejection fraction, longitudinal echocardiographic parameters can provide more sensitive insight into myocardial systolic function. In our study, changes in GDF-15 were negatively associated with MAPSE, TAPSE and S′, which indicates the association of an increase in this biomarker with less favorable changes in the longitudinal function of the left and right ventricles. This finding is in line with previous studies that showed that longitudinal dysfunction can be present in patients with preserved ejection fraction and that more advanced echocardiographic parameters, especially the assessment of longitudinal function and global longitudinal strain, can better detect septic myocardial dysfunction than standard measures of systolic function [12,13]. Recent evidence further supports the importance of comprehensive assessment of longitudinal ventricular function, particularly right ventricular function, as impairment of right ventricular performance has emerged as an independent predictor of adverse outcomes in patients with sepsis [29]. Although speckle-tracking and global longitudinal strain (GLS) were not used in our study, the association of GDF-15 with simpler longitudinal parameters may have clinical value because MAPSE, TAPSE and S′ are readily available in daily echocardiographic assessment.

Previous clinical studies of GDF-15 in sepsis have focused mainly on its prognostic value, rather than its relationship with echocardiographic parameters. Buendgens et al. showed that GDF-15 concentrations were significantly elevated in critically ill patients with sepsis and were associated with organ dysfunction, disease severity, and mortality [19]. Recent studies have further confirmed the prognostic value of GDF-15 in sepsis while highlighting the potential role as a biomarker reflecting systemic cellular stress and cardiovascular dysfunction rather than isolated myocardial injury [26]. In addition, experimental and clinical evidence suggests that serial assessment of GDF-15 may better reflect the dynamic course of septic cardiomyopathy than a single baseline measurement [30]. Contemporary reviews also emphasize that GDF-15 should be interpreted in combination with echocardiographic findings, as no single biomarker adequately captures the complex pathophysiology of septic myocardial dysfunction [27,28]. Our findings support this concept by demonstrating that dynamic changes in GDF-15 were associated with functional myocardial alterations despite the absence of consistent associations between individual GDF-15 concentrations and conventional echocardiographic parameters.

The biological plausibility of these findings is supported by previous experimental studies. GDF-15 is a cellular stress-induced cytokine that is overexpressed under conditions of inflammation, hypoxia, oxidative stress and mitochondrial dysfunction. Experimental studies have shown that GDF-15 can participate in the regulation of the inflammatory response and organ damage during sepsis, including myocardial dysfunction [20,21]. Recent evidence further suggests that GDF-15 integrates multiple pathophysiological pathways involved in septic cardiomyopathy, including immune dysregulation, mitochondrial injury, and metabolic stress, supporting its role as a biomarker reflecting the overall biological burden of myocardial dysfunction rather than isolated cardiomyocyte injury [27]. Therefore, it is possible that the increase in GDF-15 in our study is not only a marker of the severity of sepsis, but also a reflection of the activation of stress pathways associated with changes in myocardial function.

In our study, the association of GDF-15 with parameters of diastolic function, especially E/A ratio and IVRT, was observed. This mechanism may be explained by the fact that GDF-15 increases proportionally to the degree of mitochondrial stress, and the same stress causes a slower and incomplete relaxation of the left ventricle, which on the echocardiogram results in a prolongation of IVRT and a decrease in the E/A ratio. Likewise, sepsis-related inflammation causes mitochondrial dysfunction at the level of cardiomyocytes, which causes reduced ATP production and disruption of calcium transport. Landesberg et al. showed that diastolic dysfunction in severe sepsis and septic shock is associated with increased mortality, while other authors emphasized that diastolic disorders may be as important as systolic dysfunction in the assessment of septic cardiomyopathy [14,28]. The association of GDF-15 with diastolic parameters in our study therefore confirmed that this biomarker reflects not only contractile dysfunction, but also myocardial relaxation disorder during sepsis.

Unlike GDF-15, hs-TnI did not show a consistent association with echocardiographic parameters. This finding is consistent with the concept that elevated troponin concentrations in sepsis often reflect cardiomyocyte damage, increased cell membrane permeability, microcirculatory disturbances, or systemic disease severity, but do not necessarily correlate with functional echocardiographic dysfunction. Kim et al. showed that troponin I may be associated with sepsis-induced myocardial dysfunction, but its diagnostic value is not sufficient to replace echocardiographic evaluation [22]. Our results therefore support the view that hs-TnI has value as a marker of myocardial damage, but is limited as an indicator of functional myocardial changes in sepsis.

A major strength of this study is the analysis of dynamic changes in biomarkers in relation to serial echocardiographic parameters. Most previous studies of GDF-15 in sepsis have analyzed individual biomarker values and their association with disease severity or mortality, while data on the association with echocardiographic parameters are very limited. Our results suggest that a single GDF-15 value may not be sufficient to assess septic myocardial dysfunction, while changes over time may better reflect changes in myocardial functional status. Such an approach corresponds to the current understanding of septic cardiomyopathy as a reversible and time-varying syndrome. Future studies integrating serial biomarker assessment with advanced echocardiographic techniques, particularly global longitudinal strain, may further improve the characterization and early detection of septic cardiomyopathy.

Notably, although the unadjusted correlation between admission GDF-15 and MAPSE was weak and not statistically significant (rho = 0.178, *p* = 0.22), multivariable linear regression adjusted for the admission SOFA score identified an independent association between admission GDF-15 and MAPSE (β = 1.29, 95% CI 0.28–2.31, *p* = 0.01; overall model R^2^ = 0.125; F (2,46) = 3.30, *p* = 0.04). This difference between the unadjusted and adjusted analyses may indicate that illness severity influenced the crude association, with adjustment for the SOFA score revealing a relationship that was not evident in the bivariate analysis. However, this finding should be interpreted cautiously because the association was observed only for MAPSE, was not confirmed for TAPSE or other echocardiographic parameters, and the overall explanatory power of the model remained modest (adjusted R^2^ = 0.087).

This study has several limitations. First, it is a single-center study with a relatively small number of patients, which limits the generalizability of the results and reduces statistical power, especially for mortality analysis. Furthermore, echocardiographic parameters in sepsis are highly dependent on hemodynamic conditions, including volume status, afterload, vasopressor use, and mechanical ventilation, which may influence the interpretation of the findings. The study did not use advanced echocardiographic methods such as speckle-tracking analysis and global longitudinal strain, which could allow for a more precise assessment of subclinical myocardial dysfunction. The relatively small sample size limited the number of covariates included in the multivariate models to reduce the risk of overfitting. Multiple statistical testing may have increased the risk of type I error, while the lack of follow-up measurements may have introduced survivor bias. Residual confounding cannot be ruled out despite multivariate adjustment. Furthermore, the lack of a universally accepted definition of septic cardiomyopathy and the availability of only two measurement time points should be acknowledged as study limitations. Therefore, these findings should be interpreted with caution and confirmed in larger prospective studies.

In conclusion, the results of this study indicate that dynamic changes in GDF-15 are associated with changes in longitudinal systolic function and diastolic relaxation of the myocardium in patients with sepsis, and that admission GDF-15 is independently associated with longitudinal systolic function after adjustment for illness severity while hs-TnI does not show a consistent association with echocardiographic parameters. Compared to earlier studies that mainly looked at GDF-15 as a prognostic biomarker of sepsis, this study suggests a possible role as an additional biomarker of functional myocardial response. Larger, prospective and multicenter studies, ideally including GLS and standardized hemodynamic data, are needed to confirm the clinical value of serial GDF-15 determination in the evaluation of septic cardiomyopathy.

## 5. Conclusions

The results of this study showed that individual values of GDF-15 were not significantly related to most conventional echocardiographic parameters in patients with sepsis, while dynamic changes in this biomarker were associated with parameters of longitudinal systolic function and diastolic relaxation of the myocardium. In contrast, hs-TnI did not show a consistent association with echocardiographic parameters. These findings suggest the possibility that serial determination of GDF-15 may provide additional information on myocardial functional changes during sepsis, especially in combination with longitudinal echocardiographic parameters.

## Figures and Tables

**Table 1:** Demographic and clinical characteristics.

Sex [*n* (%)]	
Male	28 (56)
Female	22 (44)
Age (years)	71 (62–80)
[Median (IQR)]	
SOFA score	8 (3–12)
[Median (IQR)]	
Source of sepsis [*n* (%)]	
Respiratory tract infections (pneumonia)	30 (60)
Urinary tract infections (UTIs)	12 (24)
Cardiovascular infection (endocarditis, thrombophlebitis)	4 (8)
Biliary tract infections	3 (6)
Central nervous system infections (meningitis)	1 (2)

Data are presented as *n* (%) or median (interquartile range). Abbreviations: IQR = interquartile range; SOFA = Sequential Organ Failure Assessment; UTI = urinary tract infection.

**Table 2:** Differences in hs-TnI and GDF-15 values at admission and follow-up measurement.

	Median (Interquartile Range)		Difference ^†^	95% Confidence Interval	*p* *
	At Admission	Follow-Up			
**hs-TnI (ng/L)**	48.2 (19.5–98.0)	37.6 (17.2–180)	−5.6	−28.7 to 3.8	0.27
**GDF-15 (pg/mL)**	6812 (3192–15,100)	5917 (3292–12,563)	−522	−1787 to 132	0.08

* Wilcoxon test; ^†^ Hodges–Lehmann median difference estimate Data are presented as median (interquartile range). *p* values were calculated using the Wilcoxon signed-rank test. Differences are expressed as Hodges–Lehmann median difference estimates with 95% confidence intervals. Abbreviations: hs-TnI = high-sensitivity troponin I; GDF-15 = growth differentiation factor 15; CI = confidence interval.

**Table 3:** Differences in echocardiographic parameters between groups according to the median GDF-15 concentration at admission and during follow-up.

	Median (IQR) According to the Median GDF-15 Value		Difference ^†^	95% Confidence Interval	*p* *
**At Admission**	<6812	≥6812			
	*n* = 25	*n* = 25			
MAPSE (mm)	12 (11–14)	12 (11–14)	0	−1–2	0.56
TAPSE (mm)	22 (19–24)	22 (20–23)	1	−2–2	0.58
E wave (m/s)	0.7 (0.57–0.89)	0.74 (0.67–0.85)	0	−0.1–0.15	0.72
A wave (m/s)	0.89 (0.67–1.0)	0.80 (0.59–0.98)	−0.09	−0.26–0.1	0.41
E/A	0.77 (0.7–1.24)	0.9 (0.79–1.44)	0.1	−0.06–0.34	0.18
E′(m/s)	0.06 (0.05–0.08)	0.06 (0.05–0.08)	0	−0.01–0.01	0.96
E/E′	11.9 (9.4–13.8)	12 (8.9–15.6)	0.07	−2.5–2.9	0.93
IVRT (ms)	99 (85–105)	99 (86–112)	4	−8–14	0.58
S′ (m/s)	13 (12–15)	14 (12–17)	1	−1–3	0.38
EF Simpson BP (%)	58 (50–63)	58 (50–66)	2	−5–9	0.47
**Follow-up**	<5917	≥5917			
	*n* = 25	*n* = 25			
MAPSE (mm)	12 (10–14)	12 (9–14)	−0.8	−2–1	0.56
TAPSE (mm)	24 (19–24)	23 (19–25)	0	−3–2	0.62
E wave (m/s)	0.7 (0.6–0.9)	0.7 (0.6–0.8)	−0.05	−0.2–0.1	0.49
A wave (m/s)	0.7 (0.6–0.9)	0.8 (0.5–0.9)	0	−0.2–0.2	0.85
E/A	0.8 (0.7–1.1)	0.8 (0.6–1.4)	−0.03	−0.23–0.23	0.79
E′(m/s)	0.06 (0.05–0.07)	0.06 (0.05–0.07)	0	−0.01–0.01	0.80
E/E′	14 (10.1–16.4)	11.8 (8.3–14.8)	−1.7	−4.2–1.0	0.18
IVRT (ms)	94 (83–103)	99 (89–106)	4	−6–13	0.52
S′ (m/s)	14 (12–16)	13 (11–15)	−1	−2–1	0.60
EF Simpson BP (%)	58 (51–63)	59 (50–62)	−1	−7–5	0.70

* Mann–Whitney U test; ^†^ Hodges–Lehmann median difference estimate. Data are presented as median (interquartile range). *p* values were calculated using the Mann–Whitney U test. Differences are expressed as Hodges–Lehmann median difference estimates with 95% confidence intervals. Abbreviations: IQR = interquartile range; GDF-15 = growth differentiation factor 15; MAPSE = mitral annular plane systolic excursion; TAPSE = tricuspid annular plane systolic excursion; E = early diastolic transmitral flow velocity; A = late diastolic transmitral flow velocity; E/A = ratio of early to late transmitral flow velocity; E′ = early diastolic mitral annular velocity; E/E′ = ratio of early transmitral flow velocity to early diastolic mitral annular velocity; IVRT = isovolumetric relaxation time; S′ = peak systolic tricuspid annular velocity; EF = ejection fraction; BP = biplane; CI = confidence interval.

**Table 4:** Association between serum GDF-15 levels at admission, follow-up levels, and changes in GDF-15 concentrations and echocardiographic parameters.

	Spearman’s rho (*p*-Value) for Serum GDF-15 Concentration			
	Admission GDF-15	Follow-Up GDF-15	Change in GDF-15 (ΔGDF-15)	Relative Change in GDF-15 (ΔGDF-15)
**At admission**				
MAPSE	0.178 (0.22)	0.083 (0.57)	**−0.345 (0.02)**	**−0.320 (0.03)**
TAPSE	0.112 (0.44)	0.010 (0.95)	**−0.300 (0.03)**	−0.271 (0.06)
E wave	0.109 (0.47)	0.127 (0.40)	−0.038 (0.80)	0.048 (0.75)
A wave	−0.111 (0.51)	−0.179 (0.28)	−0.154 (0.36)	−0.147 (0.38)
E/A	**0.330 (0.04)**	**0.411 (0.01)**	0.042 (0.80)	0.160 (0.33)
E′	0.174 (0.26)	0.075 (0.63)	−0.215 (0.17)	−0.207 (0.18)
E/E′	−0.063 (0.67)	0.058 (0.70)	0.210 (0.15)	0.265 (0.07)
IVRT	−0.089 (0.56)	−0.003 (0.98)	0.296 (0.05)	0.282 (0.06)
S′	0.168 (0.25)	0.025 (0.87)	**−0.313 (0.03)**	**−0.306 (0.03)**
EF Simpson BP %	0.196 (0.17)	0.126 (0.38)	−0.271 (0.06)	−0.234 (0.10)
**Follow-up**				
MAPSE	0.112 (0.44)	−0.019 (0.90)	−0.277 (0.05)	−0.234 (0.10)
TAPSE	0.119 (0.41)	0.037 (0.80)	−0.200 (0.16)	−0.163 (0.26)
E wave	0.050 (0.73)	0.126 (0.39)	0.152 (0.30)	0.200 (0.17)
A wave	0.001 (>0.99)	−0.085 (0.61)	−0.270 (0.10)	−0.215 (0.19)
E/A	0.032 (0.85)	0.111 (0.50)	0.073 (0.66)	0.117 (0.48)
E′	0.072 (0.63)	0.071 (0.63)	0.021 (0.89)	−0.018 (0.90)
E/E′	−0.177 (0.23)	−0.135 (0.36)	0.137 (0.35)	0.158 (0.28)
IVRT	−0.099 (0.50)	0.088 (0.55)	**0.350 (0.01)**	**0.375 (0.01)**
S′	0.044 (0.76)	−0.067 (0.64)	−0.164 (0.25)	−0.188 (0.19)
EF Simpson BP %	0.143 (0.32)	0.046 (0.75)	−0.262 (0.07)	−0.242 (0.09)

Data are presented as Spearman’s correlation coefficients (rho) with corresponding *p* values. Abbreviations: GDF-15 = growth differentiation factor-15; Δ = change; MAPSE = mitral annular plane systolic excursion; TAPSE = tricuspid annular plane systolic excursion; E = early diastolic transmitral flow velocity; A = late diastolic transmitral flow velocity; E/A = ratio of early to late transmitral flow velocity; E′ = early diastolic mitral annular velocity; E/E′ = ratio of early transmitral flow velocity to early diastolic mitral annular velocity; IVRT = isovolumetric relaxation time; S′ = peak systolic tricuspid annular velocity; EF = ejection fraction; BP = biplane. Values shown in bold indicate statistically significant results.

**Table 5:** Association between serum hs-TnI levels at admission, follow-up levels, and changes in hs-TnI concentrations and echocardiographic parameters.

	Spearman’s rho (*p*-Value) for Serum hs-TnI Levels			
	Admission hs-TnI	Follow-Up hsTnI	Change in hs-TnI (ΔTnI)	Relative Change in hs-TnI (ΔTnI)
**At admission**				
MAPSE	−0.149 (0.31)	−0.149 (0.31)	0.174 (0.23)	−0.001 (>0.99)
TAPSE	0.028 (0.85)	−0.124 (0.39)	−0.119 (0.41)	−0.199 (0.17)
E wave	−0.042 (0.78)	0.192 (0.20)	0.272 (0.06)	0.264 (0.07)
A wave	−0.318 (0.05)	−0.065 (0.70)	0.145 (0.38)	0.145 (0.39)
E/A	0.082 (0.62)	0.279 (0.09)	0.192 (0.24)	0.231 (0.16)
E′	−0.131 (0.40)	0.093 (0.55)	**0.360 (0.02)**	**0.358 (0.02)**
E/E′	0.092 (0.53)	0.208 (0.16)	0.060 (0.68)	0.187 (0.20)
IVRT	0.149 (0.33)	0.002 (0.99)	−0.206 (0.17)	−0.165 (0.28)
S′	−0.017 (0.91)	0.001 (>0.99)	−0.050 (0.73)	−0.102 (0.49)
EF Simpson BP %	−0.136 (0.35)	−0.080 (0.58)	0.275 (0.05)	0.132 (0.36)
**Follow-up**				
MAPSE	−0.067 (0.65)	−0.114 (0.44)	0.098 (0.50)	−0.067 (0.65)
TAPSE	−0.005 (0.97)	−0.003 (0.98)	−0.064 (0.66)	−0.152 (0.29)
E wave	−0.222 (0.13)	−0.153 (0.30)	0.044 (0.77)	0.049 (0.74)
A wave	−0.011 (0.95)	−0.061 (0.71)	−0.163 (0.32)	−0.154 (0.35)
E/A	−0.151 (0.36)	0.032 (0.85)	0.232 (0.15)	0.211 (0.20)
E′	−0.191 (0.19)	−0.221 (0.13)	−0.078 (0.60)	−0.159 (0.28)
E/E′	0.239 (0.10)	0.256 (0.08)	0.037 (0.80)	0.105 (0.48)
IVRT	0.217 (0.13)	0.152 (0.30)	−0.125 (0.39)	−0.055 (0.71)
S′	−0.052 (0.72)	−0.165 (0.25)	−0.118 (0.41)	**−0.290 (0.04)**
EF Simpson BP %	−0.101 (0.48)	−0.087 (0.55)	0.220 (0.12)	0.045 (0.76)

Data are presented as Spearman’s correlation coefficient (rho) with corresponding *p* values. Abbreviations: hs-TnI = high-sensitivity troponin I; GDF-15 = growth differentiation factor 15; Δ = change; MAPSE = mitral annular plane systolic excursion; TAPSE = tricuspid annular plane systolic excursion; E = early diastolic transmitral flow velocity; A = late diastolic transmitral flow velocity; E/A = ratio of early to late transmitral flow velocity; E′ = early diastolic mitral annular velocity; E/E′ = ratio of early transmitral flow velocity to early diastolic mitral annular velocity; IVRT = isovolumetric relaxation time; S′ = peak systolic tricuspid annular velocity; EF = ejection fraction; BP = biplane. Values shown in bold indicate statistically significant results.

**Table 6:** Association of echocardiographic parameters with log-transformed change in GDF-15.

	β Coefficient	95% Confidence Interval	*p*-Value	R^2^
**At admission**				
MAPSE	−0.02	−0.04–0.006	0.14	0.05
E wave	−0.01	−0.32–0.31	0.99	0.001
E′	−2.09	−5.54–1.35	0.23	0.04
EF Simpson BP %	−0.004	−0.009–0.001	0.15	0.04
**Follow-up**				
MAPSE	−0.02	−0.04–0.004	0.10	0.06
E wave	0.13	−0.18–0.44	0.40	0.02
E′	0.65	−1.69–2.98	0.58	0.007
EF Simpson BP %	−0.005	−0.01–0.001	0.11	0.05

Data are presented as β coefficients with corresponding 95% confidence intervals, *p* values, and R^2^ values. Abbreviations: GDF-15 = growth differentiation factor 15;MAPSE = mitral annular plane systolic excursion; E = early diastolic transmitral flow velocity; E′ = early diastolic mitral annular velocity; EF = ejection fraction; BP = biplane; CI = confidence interval; β = regression coefficient; R^2^ = coefficient of determination. Values shown in bold indicate statistically significant results.

**Table 7:** Association of echocardiographic parameters with the log-transformed relative change in hs-TnI.

	β Coefficient	95% Confidence Interval	*p*-Value	R^2^
**At admission**				
MAPSE	−0.07	−0.17–0.03	0.14	0.05
E wave	0.14	−1.21–1.49	0.83	0.001
E′	−2.99	−17.4–11.4	0.68	0.004
EF Simpson BP %	−0.01	−0.04–0.01	0.29	0.02
**Follow-up**				
MAPSE	−0.01	−0.12–0.09	0.81	0.001
E wave	−0.92	−2.23–0.38	0.16	0.04
E′	1.37	−9.5–12.3	0.80	0.001
EF Simpson BP %	−0.01	−0.04–0.02	0.45	0.01

Data are presented as β coefficients with corresponding 95% confidence intervals, *p* values, and R^2^ values. Abbreviations: hs-TnI = high-sensitivity troponin I; MAPSE = mitral annular plane systolic excursion; E = early diastolic transmitral flow velocity; E′ = early diastolic mitral annular velocity; EF = ejection fraction; BP = biplane; CI = confidence interval; β = regression coefficient; R^2^ = coefficient of determination. Values shown in bold indicate statistically significant results.

**Table 8:** Associations of SOFA score and log-transformed GDF-15 concentration with MAPSE and TAPSE at admission and at control (multivariate linear regression).

Dependent Variable	Predictor	β	95% Confidence Interval	*p* *	Regression Model
**Admission**					
MAPSE	log(GDF-15) at admission	1.29	0.28–2.31	**0.01**	R^2^ = 0.125; R^2^_cor_ = 0.087
	SOFA at admission	−0.07	−0.25–0.11	0.44	F _(2,46)_ = 3.3; *p* = 0.04
TAPSE	log(GDF-15) on admission	−0.19	−1.97–1.59	0.83	R^2^ = 0.001; R^2^_cor_ = −0.04
	SOFA at admission	0.02	−0.30–0.34	0.91	F _(2,47)_ = 0.3; *p* = 0.97
**Follow-up**					
MAPSE	log(GDF-15) at follow−up	0.23	−1.08–1.54	0.73	R^2^ = 0.003; R^2^_cor_ = −0.04
	SOFA at admission	−0.02	−0.20–0.16	0.86	F _(2,46)_ = 0.08; *p* = 0.92
TAPSE	log(GDF-15) at follow−up	−0.15	−1.94–1.64	0.87	R^2^ = 0.001; R^2^_cor_ = −0.04
	SOFA at admission	−0.01	−0.25–0.23	0.94	F _(2,47)_ = 0.02; *p* = 0.98

Data are presented as regression coefficients with corresponding 95% confidence intervals. Abbreviations: SOFA = Sequential Organ Failure Assessment; GDF-15 = growth differentiation factor 15; MAPSE = mitral annular plane systolic excursion; TAPSE = tricuspid annular plane systolic excursion; CI = confidence interval; β = regression coefficient; R^2^ = coefficient of determination; R^2^_cor_ = adjusted coefficient of determination; F = F statistic. Values shown in bold indicate statistically significant results. *p* * refers to multivariate regression.

**Table 9:** Association of SOFA score and hs-TnI with MAPSE and TAPSE at admission and control (multivariate linear regression).

Dependent Variable	Predictor	β	95% Confidence Interval	*p* *	Regression Model
**Admission**					
MAPSE	log(hsTnI) on admission	−0.61	−1.44–0.22	0.15	R^2^ = 0.046; R^2^_cor_ = 0.005
	SOFA at admission	−0.02	−0.20–0.17	0.85	F _(2,46)_ = 1.1; *p* = 0.34
TAPSE	log(hsTnI) on admission	0.54	−0.84–1.91	0.44	R^2^ = 0.013; R^2^_cor_ = −0.03
	SOFA at admission	0.007	−0.30–0.31	0.96	F _(2,47)_ = 0.31; *p* = 0.74
**Follow-up**					
MAPSE	log(hsTnI) at follow-up	0.40	−0.84–1.64	0.52	R^2^ = 0.01; R^2^_cor_ = −0.03
	SOFA at admission	−0.02	−0.20–0.16	0.82	F _(2,46)_ = 0.23; *p* = 0.79
TAPSE	log(hsTnI) at follow-up	0.49	−1.19–2.18	0.56	R^2^ = 0.007; R^2^_cor_ = −0.04
	SOFA at admission	−0.01	−0.25–0.23	0.92	F _(2,47)_ = 0.12; *p* = 0.84

Data are presented as regression coefficients with corresponding 95% confidence intervals. Abbreviations: SOFA = Sequential Organ Failure Assessment; hs-TnI = high-sensitivity troponin I; MAPSE = mitral annular plane systolic excursion; TAPSE = tricuspid annular plane systolic excursion; CI = confidence interval; β = regression coefficient; R^2^ = coefficient of determination; R^2^_cor_ = adjusted coefficient of determination; F = F statistic. *p* * refers to multivariate regression.

## Data Availability

The original contributions presented in this study are included in the article. Further inquiries can be directed to the corresponding authors.

## Abbreviations

A	late diastolic transmitral flow velocity
ATP	adenosine triphosphate
BP	biplane
CI	confidence interval
E	early diastolic transmitral flow velocity
E′	early diastolic mitral annular velocity
E/A	ratio of early to late transmitral flow velocity
E/E′	ratio of early transmitral flow velocity to early diastolic mitral annular velocity
ECLIA	electrochemiluminescent immunoassay
EF	ejection fraction
F	F statistic
GDF-15	growth differentiation factor-15
GLS	global longitudinal strain
GPX4	glutathione peroxidase 4
hs-TnI	high-sensitivity troponin I
ICU	intensive care unit
IQR	interquartile range
IVRT	isovolumetric relaxation time
LVEF	left ventricular ejection fraction
MAP	mean arterial pressure
MAPSE	mitral annular plane systolic excursion
MIC-1	macrophage inhibitory cytokine-1
R^2^	coefficient of determination
R^2^_cor_	adjusted coefficient of determination
SOCS1	suppressor of cytokine signaling 1
SOFA	Sequential Organ Failure Assessment
S′	peak systolic tricuspid annular velocity
TAPSE	tricuspid annular plane systolic excursion
TGF-β	transforming growth factor beta
UTI	urinary tract infection
β	regression coefficient
